# Predicting the Role of DNA Polymerase β Alone or with *KRAS* Mutations in Advanced NSCLC Patients Receiving Platinum-Based Chemotherapy

**DOI:** 10.3390/jcm9082438

**Published:** 2020-07-30

**Authors:** Maria Francesca Alvisi, Monica Ganzinelli, Helena Linardou, Elisa Caiola, Giuseppe Lo Russo, Fabiana Letizia Cecere, Anna Cecilia Bettini, Amanda Psyrri, Michele Milella, Eliana Rulli, Alessandra Fabbri, Marcella De Maglie, Pierpaolo Romanelli, Samuel Murray, Gloriana Ndembe, Massimo Broggini, Marina Chiara Garassino, Mirko Marabese

**Affiliations:** 1 Laboratory of Methodology for Clinical Research, Department of Oncology, Istituto di Ricerche Farmacologiche Mario Negri IRCCS, 20156 Milan, Italy; mariafrancesca.alvisi@marionegri.it (M.F.A.); eliana.rulli@marionegri.it (E.R.); 2 Unit of Thoracic Oncology, Medical Oncology Department 1, Fondazione IRCCS Istituto Nazionale dei Tumori, 20133 Milan, Italy; monica.ganzinelli@istitutotumori.mi.it (M.G.); Giuseppe.LoRusso@istitutotumori.mi.it (G.L.R.); marina.garassino@istitutotumori.mi.it (M.C.G.); 3 4th Oncology Department, Metropolitan Hospital, 18547 Athens, Greece; elinardou@otenet.gr; 4 Laboratory of Molecular Pharmacology, Department of Oncology, Istituto di Ricerche Farmacologiche Mario Negri IRCCS, 20156 Milan, Italy; elisa.caiola@marionegri.it (E.C.); gloriana.ndembe@marionegri.it (G.N.); 5 Division of Medical Oncology 1, IRCCS Regina Elena National Cancer Institute, 00144 Rome, Italy; fabianacecere@gmail.com; 6 UO Oncologia Medica, ASST Papa Giovanni XXIII, 24127 Bergamo, Italy; abettini@asst-pg23.it; 7 Section of Oncology, Department of Internal Medicine, Attikon Hospital, National Kapodistrian University of Athens, 12462 Athens, Greece; psyrri237@yahoo.com; 8 Department of Medicine, Section of Medical Oncology, University and Hospital Trust of Verona, 37126 Verona, Italy; michele.milella@aovr.veneto.it; 9 Department of Pathology and Laboratory Medicine, Fondazione IRCCS Istituto Nazionale dei Tumori, 20133 Milan, Italy; Alessandra.fabbri@istitutotumori.mi.it; 10 Mouse & Animal Pathology Lab, Fondazione Filarete, 20139 Milan, Italy; marcellademaglie@libero.it (M.D.M.); pierpaolo.romanelli.medvet@gmail.com (P.R.); 11 Department of Veterinary Medicine, University of Milan, 20122 Milan, Italy; 12 Biomarker Solutions Ltd., London EC1V 2NX, UK; smgenedb@gmail.com

**Keywords:** NSCLC, KRAS, DNA polymerase beta, platinum-based first-line

## Abstract

Clinical data suggest that only a subgroup of non-small cell lung cancer (NSCLC) patients has long-term benefits after front-line platinum-based therapy. We prospectively investigate whether KRAS status and DNA polymerase β expression could help identify patients responding to platinum compounds. Prospectively enrolled, advanced NSCLC patients treated with a first-line regimen containing platinum were genotyped for KRAS and centrally evaluated for DNA polymerase β expression. Overall survival (OS), progression-free survival (PFS), and the objective response rate (ORR) were recorded. Patients with KRAS mutations had worse OS (hazard ratio (HR): 1.37, 95% confidence interval (95% CI): 0.70–2.27). Negative DNA polymerase β staining identified a subgroup with worse OS than patients expressing the protein (HR: 1.43, 95% CI: 0.57–3.57). The addition of KRAS to the analyses further worsened the prognosis of patients with negative DNA polymerase β staining (HR: 1.67, 95% CI: 0.52–5.56). DNA polymerase β did not influence PFS and ORR. KRAS may have a negative role in platinum-based therapy responses in NSCLC, but its impact is limited. DNA polymerase β, when not expressed, might indicate a group of patients with poor outcomes. KRAS mutations in tumors not expressing DNA polymerase β further worsens survival. Therefore, these two biomarkers together might well identify patients for whom alternatives to platinum-based chemotherapy should be used.

## 1. Introduction

Over the last 40 years, several million lung cancer patients have received platinum-based regimens, and despite the clinical use of an impressive variety of targeted agents, these drugs are still one of the main therapeutic options for certain patients [1]. Platinum compounds are also the best choice in first-line immunotherapy combinations [2]. However, despite the good impact of platinum-based therapies, only a small proportion of patients have durable benefits [3]. Therefore, biomarkers to explain the resistance mechanisms to platinum compounds are urgently needed.

*KRAS* mutations have long been considered potential biomarkers to predict the outcome of platinum-based chemotherapy in NSCLC [4]. The TAILOR trial data shed light on the possibility that there was a small negative prognostic effect of *KRAS* mutations in advanced NSCLC patients treated with a platinum-based doublet when EGFR-mutant patients were excluded from the analysis [5]. 

Platinum adducts are repaired by different DNA repair systems. The Fanconi anemia (FA) pathway is thought to coordinate these systems, including homologous recombination (HR), nucleotide excision repair (NER), and translesion synthesis (TLS) repair [6,7]. Other DNA repair systems, such as base excision repair (BER), are involved in cisplatin-induced DNA damage, but so far, they have been assigned only a marginal role in repairing this damage [8].

Our group recently reported in a preclinical study that DNA polymerase β, an important component of the BER pathway, could be involved in platinum-based chemotherapy responses. Our results suggested a different pattern of sensitivity/resistance to cisplatin, dependent on *KRAS* mutational status [9].

The present work explores whether DNA polymerase β, alone or in combination with *KRAS* mutational status, can identify tumors with different abilities to respond to platinum compounds. This is the first study to prospectively assess the combined role of the selected biomarkers to identify patients who could benefit from platinum-based therapy.

## 2. Material and Methods

### 2.1. Study Population and Samples

The Fondazione IRCCS Istituto Nazionale dei Tumori (Milan, Italy), the Regina Elena National Cancer Institute (Rome, Italy), the Hospital Papa Giovanni XXIII (Bergamo, Italy), and the Metropolitan and Attikon Hospitals (Athens, Greece) were the centers involved. Consecutive patients with metastatic NSCLC who received platinum-based chemotherapy in combination with either vinorelbine, gemcitabine, or pemetrexed, according to the physician’s choice, as first-line therapy between February 2014 and April 2017 were included in the BioRaRe prospective multicenter trial. 

All patients had an Eastern Cooperative Oncology Group (ECOG) Performance Status (PS) between 0 and 2 and were at least 18 years of age. Exclusion criteria included any evidence of serious comorbidities that the investigator judged as a contraindication to the participation in the study, pregnancy, and breast-feeding.

Patients evaluable for tumor response according to the RECIST 1.1 criteria were examined, and their demographics and clinical and pathological characteristics were retrieved. E-CRF and medical records were used to collect data. 

The study was approved by the Fondazione IRCCS Istituto Nazionale dei Tumori Institutional Review Board (INT18/13) and conducted according to the Declaration of Helsinki ethical principles for medical research involving human subjects. All patients gave signed written informed consent.

### 2.2. Mutational Analysis

*KRAS* mutational status was determined by Sanger sequencing at each center, following the protocol already used in a clinical trial by our group [10]. Briefly, DNA extraction was performed on histological tumor specimens by using standard phenol–chloroform procedure after macro/microdissection in order to recovery most of the cancer cells and to reduce contamination by normal ones. DNA preparations were verified for their concentration and quality by spectrophotometric measurement. Genomic DNAs were amplified by polymerase chain reaction (PCR) using high-fidelity Taq polymerase and specific primers encompassing intronic regions for KRAS exons 2–4. PCR products were then analyzed electrophoretically on agarose gel, and automated bidirectional sequencing was performed using BigDye Terminator chemistry. Sequences were then automatically compared with wild-type KRAS gene profiles by software analysis to assess the presence of possible mutations.

### 2.3. Immunohistochemical Analysis (IHC)

IHC was done centrally on single slides at the Fondazione Filarete, as previously reported [11]. Sections were immune-stained with anti-DNA polymerase β antibody ab26343 (Abcam, Cambridge, UK), and incubated with biotinylated secondary goat anti-rabbit antibody (VC-BA-1000-MM15, Vector Laboratories, Burlingame, CA, USA). Sections were labeled by the avidin–biotin–peroxidase (ABC) procedure with a commercial immunoperoxidase kit (VECTASTAIN Elite ABC-Peroxidase Kit Standard, VC-PK-6100-KI01, Vector Laboratories, Burlingame, CA, USA). The immune reaction was visualized with 3,3′-diaminobenzidine peroxidase DAB substrate kit (VC-SK-4100-KI01, Vector Laboratories, Burlingame, CA, USA) substrate and sections were counterstained with Mayer’s hematoxylin. Figure S1 shows representative images of negative and positive DNA polymerase β staining.

A semiquantitative H-score (percentage of positive tumoral cells x intensity: 0 = negative, 1 = slight, 2 = moderate, 3 = strong) was calculated independently by two pathologists. In case of disagreement, a third opinion was requested.

### 2.4. Outcomes

The primary outcome of the study was progression-free survival (PFS). Secondary outcomes were objective response rate (ORR) and overall survival (OS). PFS was defined as the time from the start of the platinum-based first-line therapy to the date of progression or death from any cause, whichever came first. ORR was defined as the proportion of patients with a complete or partial response to treatment. OS was defined as the time from the platinum-based first-line therapy to the date of death from any cause.

### 2.5. Statistical Methods

Chi-squared and Kruskal–Wallis tests were used to analyze the relations between the DNA polymerase β H-score (Polβ) and categorical clinical variables. The Spearman correlation coefficient was used to measure the correlation between Polβ and continuous clinical variables. Polβ was analyzed as a continuous and dichotomous variable (Polβ = 0 as negative and Polβ > 0 as positive). 

Patients who had not died or had no disease progression were censored at their last available information on status. Survival curves were calculated with the Kaplan–Meier method and tested by the log-rank test. Cox proportional hazard models were used to analyze the impact of DNA polymerase β on PFS and OS, adjusting for clinical and pathological characteristics such as ECOG-PS, age, histology, smoking, therapy, and, only for OS, immunotherapy. Results were expressed as hazard ratios (HRs) with their 95% confidence intervals (95% CIs). 

The impact of DNA polymerase β on ORR was analyzed with logistic regression models and expressed as odds ratios (ORs) with their 95% CIs, while for dichotomized analysis, the chi-square test was used. A subgroup analysis was done for patients with both Polβ and KRAS mutational status available. 

All statistical tests were two-sided, and *p* < 0.05 was considered statistically significant. Statistical analyses were done using SAS version 9.4 (SAS Institute, Cary, NC, USA).

## 3. Results 

**Figure 1:**
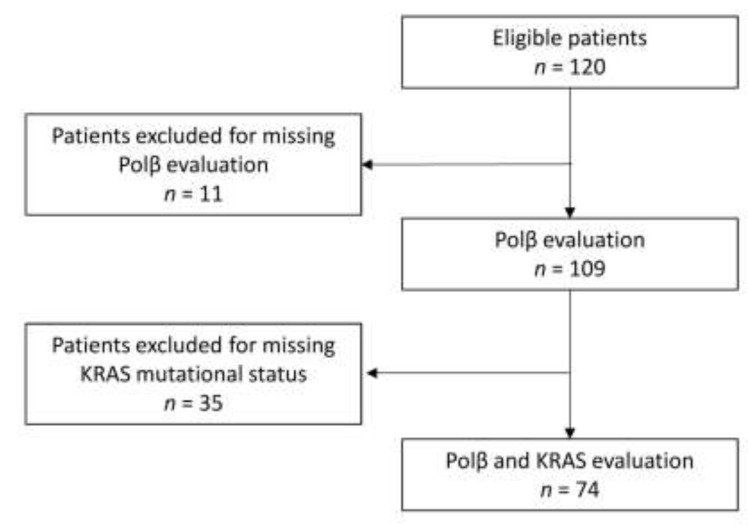
CONSORT diagram showing the flow of participants.

Of the 120 patients registered in the trial with material available, 109 had a DNA polymerase β H-score (Polβ) and 74 had both Polβ and *KRAS* mutational status. Figure 1 reports the flowchart of the study.

The main demographic characteristics of the population (*n* = 109) and the relationships between characteristics and Polβ are reported in Table 1.

### 3.1. Progression-Free Survival

The median PFS was, respectively, 5.9 and 7.2 months in the mutated (mut) and wild-type (wt) KRAS groups (adjusted HR mut vs. wt: 1.09, 95% CI: 0.56–2.08, *p* = 0.815).

**Figure 2:**
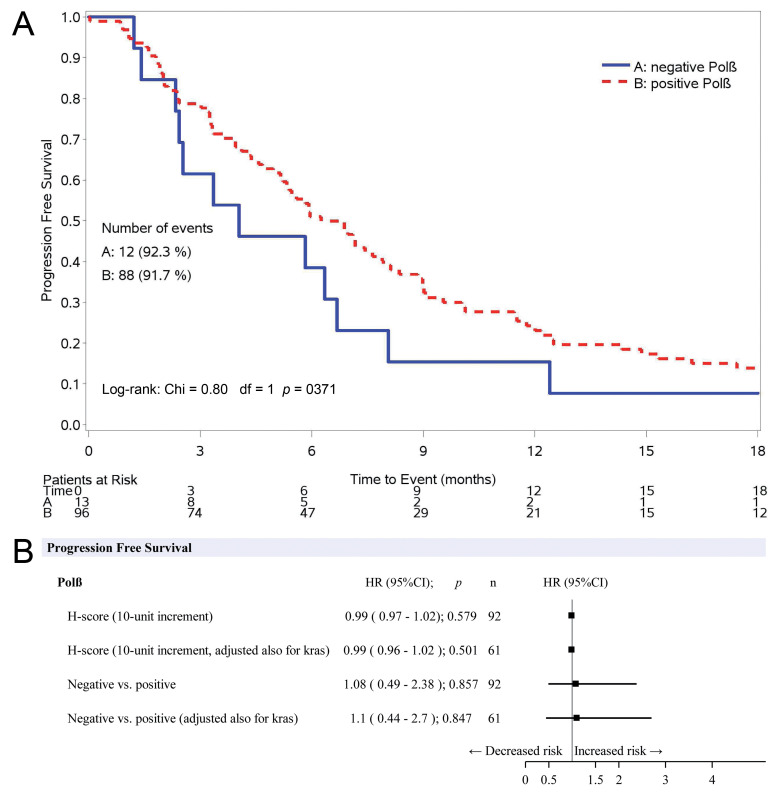
(**A**) Kaplan–Meier curves for PFS according to the positive or negative DNA polymerase β staining. (**B**) Effect of KRAS status on the relationship between Polβ and PFS adjusted for ECOG-PS, age, histology, smoking, and therapy.

Polβ, considered a continuous variable, did not have any significant impact on PFS in a multivariable Cox model. HR was 0.99 for each 10-unit increment of the score, with 95% CI 0.97–1.02 and *p* = 0.579. The inclusion of KRAS mutational status in the statistical model did not modify the impact of Polβ on progression or death risk (HR: 0.99, 95% CI: 0.96–1.02, *p* = 0.501). Considering Polβ as a dichotomous variable, median PFS were, respectively, 4 and 6.3 months for negative (neg) and positive (pos) staining. The absence or presence of DNA polymerase β had no impact on the risk of PFS, considering the multivariable models, either including KRAS status or not in the analysis (HR pos vs. neg: 1.10, 95% CI: 0.44–2.70, *p* = 0.847; HR pos vs. neg: 1.08, 95% CI: 0.49–2.38, *p* = 0.857). Detailed results of the multivariable analysis for PFS are reported in Table 2, and the Kaplan–Meier curves for PFS are shown in Figure 2A. The forest plot in Figure 2B graphically shows the effect of KRAS status on the relationship between Polβ and PFS.

### 3.2. Overall Survival

The median OS was, respectively, 12.4 and 20.5 months in the mutated and wild-type KRAS groups (adjusted HR mut vs. wt: 1.27, 95% CI: 0.70–2.27, *p* = 0.441).

Polβ, analyzed as a continuous variable, had no impact on survival in the multivariable models including KRAS status or not (HR: 0.99, 95% CI: 0.96–1.01, *p* = 0.39; HR = 0.99, 95% CI: 0.95–1.02, *p*-value = 0.388).

**Figure 3:**
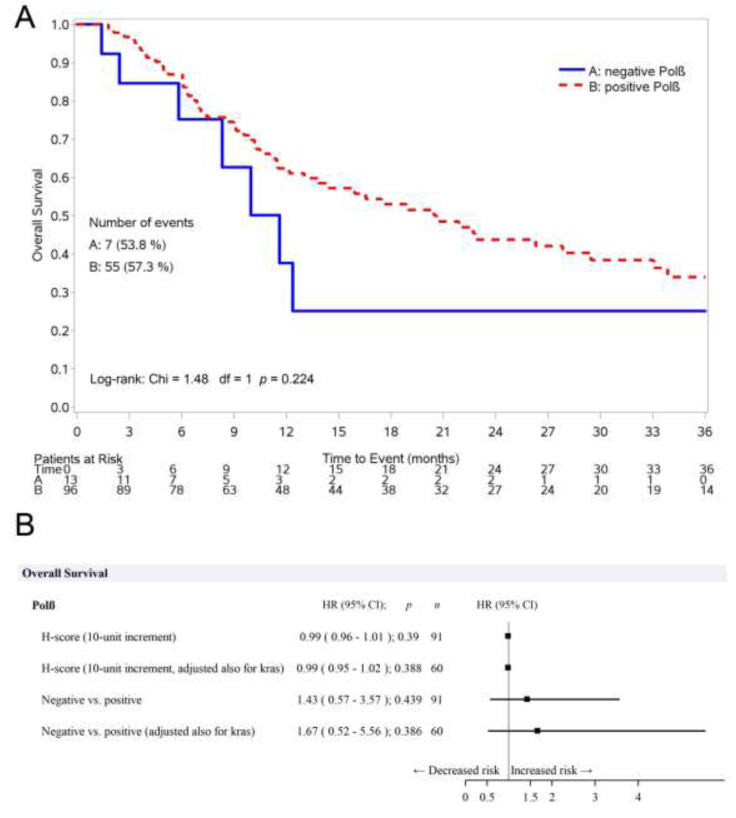
(**A**) Kaplan–Meier curves for OS, according to the positive or negative DNA polymerase β staining. (**B**) Effect of KRAS status on the relationship between Polβ and PFS adjusted for ECOG-PS, age, histology, smoking, therapy, and immunotherapy.

Patients who were negative for DNA polymerase β staining had a median OS of 11.6 months compared to 20.6 months in the positive group. The absence of DNA polymerase β caused a worse but not statistically significant OS compared to DNA polymerase β-expressing patients (HR pos vs. neg: 1.43, 95% CI: 0.57–3.57, *p* = 0.439). With the inclusion of KRAS mutational status in the statistical model, the effect on survival with Polβ was stronger (HR pos vs. neg: 1.67, 95% CI: 0.52–5.56, *p* = 0.386). The results of the multivariate analyses for OS are reported in Table 3. Kaplan–Meier curves for OS, reported in Figure 3A,B, show the effect of KRAS status on the relationship between Polβ and OS.

### 3.3. Overall Response Rate

There were no differences between the DNA polymerase β negative and positive staining groups, or among different Polβ as a continuous variable in ORR to platinum-based first-line therapy (Table 4).

## 4. Discussion

KRAS mutations have often been investigated as possible biomarkers for selecting chemotherapy, but results have varied, casting doubt on the true utility of this protein. In a previously published randomized prospective trial from our group, an analysis of 247 patients showed that those carrying KRAS mutations and treated with a first-line platinum-based regimen had worse PFS than patients with wild-type KRAS [5]. The present study detected a not-statistically-significant effect for OS, KRAS-mutated patients having a worse prognosis than KRAS wild-type patients. A possible explanation, although the trend is in line with previous observations, is that the statistical power of this cohort of patients was half that in our earlier study, where KRAS status was significantly associated with survival. On the other hand, the LACE-Bio pooled analysis, including data of 1543 patients participating in four clinical trials, showed that there is no difference in terms of outcomes in early-stage lung cancer patients with either wild-type or mutated KRAS [12]. Our different result may suggest that KRAS mutations could play different roles in early and advanced disease. In advanced stages, KRAS could be a condition necessary, but not sufficient, to explain a more aggressive phenotype.

There is preclinical evidence that KRAS and its mutated versions modulate DNA repair, hence the cellular response to genotoxic agents. Oncogenic RAS can inactivate BRCA-1 dependent homologous recombination (HR) by favoring the dissociation of BRCA-1 from chromatin [13]. Moreover, activated KRAS can suppress the expression of DNA repair genes (including BRCA1, BRCA2, EXO-1, and TP53) [14]. In leukemic cells, mutant KRAS promoted the upregulation of components of the alternative nonhomologous end-joining (NHEJ) pathway, such as DNA ligase IIIα, PARP1, and XRCC1, and the inhibition of the alternative NHEJ pathway selectively sensitized KRAS-mutated cells to chemotherapy [15].

Our group also suggested KRAS-dependent specific alterations in the BER system, where we found DNA polymerase β as a possible selection factor. We demonstrated at the preclinical level that DNA polymerase β could play a role in the response to cisplatin-based chemotherapy, and the data indicated a pattern of sensitivity or resistance depending on the KRAS mutational status [9]. These findings support the hypothesis that the combination of mutant-KRAS status with DNA repair could be a predictive biomarker for response to platinum-based therapy. 

On the basis of these assumptions, we planned a translational study to clinically validate KRAS and DNA polymerase β as “biomarkers” for poor response and outcome to platinum-based first-line chemotherapy. We investigated DNA polymerase β as a possible selection marker, alone or in combination with KRAS status. DNA polymerase β expression, summarized in the H-score and considered as a continuous variable, was meaningless to both PFS and OS, alone or with KRAS.

When we compared negative or positive DNA polymerase β staining patients, we detected an interesting, though not statistically significant, difference: OS patients negative for DNA polymerase β staining had worse outcomes than the positive staining group. This result was confirmed even when KRAS status was considered in the analysis. 

These data, although interesting and calling for further analysis, are not supported by the literature, where DNA polymerase β upregulation was described as causing resistance to cisplatin in an ovarian cancer model [16]. In a colorectal cancer model expressing high levels of DNA polymerase β, cisplatin was ineffective compared to the same model in which DNA polymerase β was downregulated. In the same paper, 5-year OS curves showed that patients with high DNA polymerase β expression had a significantly poorer prognosis than those with low expression [17]. However, DNA polymerase β has been investigated as a selection marker in very few, only retrospective studies, and our is the first attempt to investigate it, prospectively, in NSCLC.

A recent report suggests that if cells are not able to repair DNA single-strand break lesions through BER (as should be the case here for cells negative for DNA polymerase β), these lesions are channeled to the HR system [18]. We do not know whether this is also true for cisplatin-induced DNA lesions and whether these patients have HR alterations, but it does suggest an intriguing explanation for the worse outcome observed in DNA polymerase β-negative patients.

To our knowledge, this is the first investigation of the role and value of DNA polymerase β, alone or in combination with KRAS status, as a marker of response to platinum-based therapy in NSCLC. Besides the results, this paper also stimulates the idea to further investigate the combination of biomarkers that indicate how different biological pathways coexist or work together in those scenarios, where no single biomarker has been shown to have strong value.

In conclusion, KRAS may have a negative role in platinum-based therapy responses in NSCLC, but its impact is limited. The absence of DNA polymerase β might indicate a group of patients with poor outcomes compared to patients positively staining for this protein. In addition, a mutated form of KRAS in tumors not expressing DNA polymerase β further worsens survival. Therefore, these two biomarkers together might well identify patients for whom alternatives to platinum-based chemotherapy should be used.

## Figures and Tables

**Table 1:** Baseline characteristics of patients (*n* = 109) and their relation with Polβ as a continuous or dichotomous variable. Pos, positive; neg, negative.

		*n* (%)		*p*	*p*
				Polβ Continuous	Polβ pos vs. neg
Age of diagnosis	Median(Q1–Q3)	66.8 (60.0–71.4)		0.448 *	0.788 ^†^
	Missing	3			
Gender	Male	70	65.4	0.717 ^†^	0.366 **
	Female	37	34.6		
	Missing	2			
ECOG-PS	0	78	81.3	0.157 ^†^	0.443 **
	1	17	17.7		
	2	1	1		
	Missing	13			
Smoking	Never	21	20	0.618 ^†^	1.000 **
	Former smokers	42	40		
	Smokers	42	40		
	Missing	4			
Stage at diagnosis	*IIIB*	28	26.2	0.038 ^†^	0.507 **
	*IV*	79	73.8		
	Missing	2			
Histotype	Adenocarcinoma	90	82.6	0.291 ^†^	0.184 **
	Squamous	17	15.6		
	Other	2	1.8		
Platinum-based therapy	Cisplatin	33	34.7	0.726 ^†^	0.486 **
	Carboplatin	62	65.3		
	Missing	14			
Immunotherapy	No	62	58.5	0.248 ^†^	0.352 **
	Yes	44	41.5		
	Missing	3			
Polβ	Median(Q1-Q3)	160.0 (60.0–200.0)		-	-
	negative	13	11.9	-	-
	positive	96	88.1		
KRAS	Mutated	35	47.3	0.053 ^†^	0.125 **
	Wild-Type	39	52.7		
	Missing	35			

At a median follow-up of 18.8 months (Q1–Q3: 8.3–48.9), there were 90 progressions, 62 deaths, and 100 deaths or progressions. Q1–Q3: first–third quartile, pos: positive, neg: negative, ^†^: Kruskal–Wallis test, *: Spearman correlation, **: Fisher test.

**Table 2:** Multivariable Cox models adjusted for ECOG-PS, age, histology, smoking, and therapy for progression-free survival, considering Polβ continuous or Polβ positive vs. negative. Pos, positive; neg, negative.

		Polβ Continuous		Polβ pos vs. neg	
		HR (95% CI)	*p*	HR (95% CI)	*p*
**Polβ (10-unit increment)**		0.99 (0.97–1.02)	0.579	-	-
**Polβ**					
Positive		-	-	reference	
Negative		-	-	1.07 (0.49–2.38)	0.857
**Age at metastasis diagnosis (5 years increment)**		0.82 (0.72–0.94)	0.005	0.82 (0.72–0.95)	0.006
**Histology**					
Adenocarcinoma		reference		reference	
Squamous		1.10 (0.60–2.03)	0.755	1.12 (0.60–2.10)	0.728
Nos or other		2.26 (0.27–18.6)	0.449	2.34 (0.28–19.5)	0.432
**Smoke**					
Never		reference		reference	
Previous		1.25 (0.69–2.26)	0.463	1.27 (0.70–2.29)	0.434
Current		0.79 (0.40–1.56)	0.495	0.81 (0.41–1.59)	0.543
**ECOG-PS**		1.51 (0.78–2.94)	0.223	1.51 (0.76–3.00)	0.244
**Therapy**					
Cisplatin		reference		reference	
Carboplatin		1.73 (1.02–2.92)	0.041	1.69 (1.01–2.85)	0.046

**Table 3:** Multivariable Cox models adjusted for ECOG-PS, age, histology, smoking, therapy, and immunotherapy for OS, considering Polβ continuous and Polβ positive or negative. Pos: positive, neg: negative.

	Polβ Continuous		Polβ pos vs. neg	
	HR (95% CI)	*p*	HR (95% CI)	*p*
**Polβ (10-unit increment)**	0.99 (0.96–1.01)	0.390	-	-
**Polβ**				
Positive	-	-	reference	
Negative	-	-	1.43 (0.57–3.57)	0.439
**Age at metastasis diagnosis (5 years increments)**	0.87 (0.75–1.01)	0.066	0.87 (0.75–1.01)	0.065
**Histology**				
Adenocarcinoma	reference		reference	
Squamous	0.94 (0.46–1.95)	0.877	0.98 (0.47–2.06)	0.960
Nos or other	7.67 (0.83–70.6)	0.072	8.86 (0.94–83.3)	0.056
**Smoke**				
Never	reference		reference	
Previous	2.65 (1.15–6.12)	0.022	2.76 (1.21–6.30)	0.016
Current	1.58 (0.61–4.11)	0.350	1.63 (0.63–4.26)	0.316
**ECOG-PS**	1.18 (0.48–2.90)	0.724	1.12 (0.44–2.87)	0.812
**Therapy**				
Cisplatin	reference		reference	
Carboplatin	1.74 (0.94–3.21)	0.075	1.70 (0.93–3.12)	0.084
**Immunotherapy**				
No	reference		reference	
Yes	0.57 (0.32–1.03)	0.063	0.55 (0.31–0.98)	0.041

**Table 4:** Objective response rates by DNA Polymerase β H-score (Polβ). CR, complete response; PR, partial response; SD, stable disease; PD, progressive disease.

	Polβ neg *n* = 12	Polβ pos *n* = 64	Chi-Squared Test	Logistic Regression Model
CR + PR −*n* (%)	4 (33.3)	23 (35.9)	Chi = 0.03	OR = 1.002
95% CI	34.9–90.1	51.1–75.7	Df = 1	95%CI = 0.997–1.006
SD + PD −*n* (%)	8 (66.7)	41 (64.1)	*p* = 0.864	*p* = 0.505
95% CI	9.9–65.1	24.3–48.9		

## Supplementary Materials

The following are available online at https://www.mdpi.com/2077-0383/9/8/2438/s1, Figure S1: Negative and positive DNA polymerase β staining tissues magnified 400X.
