# Supplementary figures and images for: Predicting the Role of DNA Polymerase β Alone or with KRAS Mutations in Advanced NSCLC Patients Receiving Platinum-Based Chemotherapy
Source: J Clin Med. 2020 Jul 30;9(8):2438. doi: 10.3390/jcm9082438 (PMC7465625; doi:10.3390/jcm9082438)

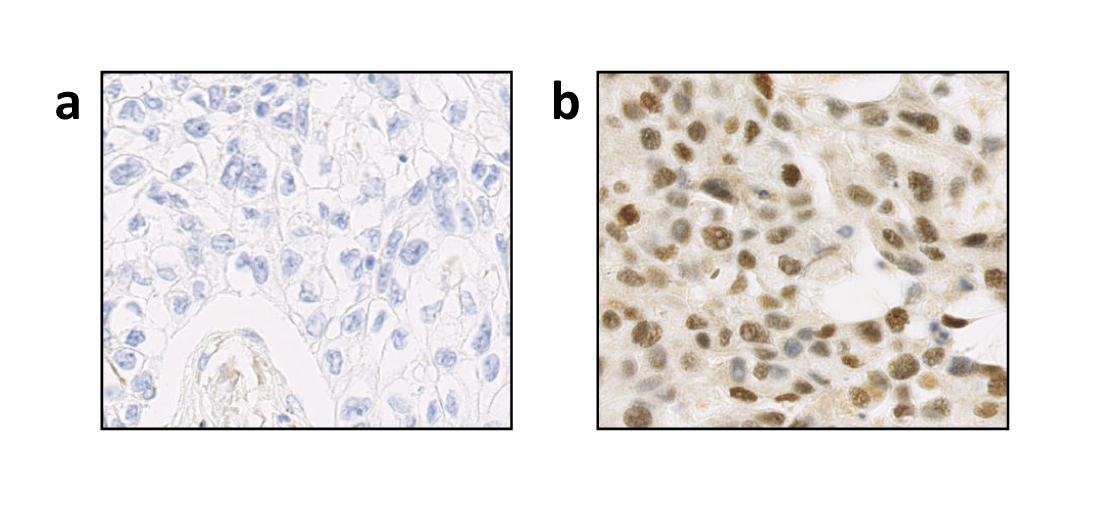

Supplement: Supplementary file 1 [file jcm-09-02438-s001.zip › jcm-863642-supplementary.tif]
